# Supplementary material for: Stent for life initiative in Portugal: Progress through years and Covid-19 impact
Source: PLoS One. 2023 Oct 25;18(10):e0284915. doi: 10.1371/journal.pone.0284915 (PMC10599499; doi:10.1371/journal.pone.0284915)
Supplement: Attachment — Submitted filename: Rebuttal Letter.docx [file pone.0284915.s001.docx]

Rebuttal Letter

Dear Editor

On behalf of my co-authors I would like to thank the opportunity to provide clarifications that were not clearly detailed in the previous version of the manuscript.

All comments and suggestions from the editors and reviewers were considered and modifications were made as suggested to clarify all aspects that were less clear.

Concerning Journal Requirements:

*1. Please ensure that your manuscript meets PLOS ONE's style requirements, including those for file naming.*

Revised and corrected.

*2. Please provide additional details regarding participant consent. In the ethics statement in the Methods and online submission information, please ensure that you have specified what type you obtained (for instance, written or verbal, and if verbal, how it was documented and witnessed).*

Information has been completed and detailed

*3. We note that you have stated that you will provide repository information for your data at acceptance. Should your manuscript be accepted for publication, we will hold it until you provide the relevant accession numbers or DOIs necessary to access your data. If you wish to make changes to your Data Availability statement, please describe these changes in your cover letter and we will update your Data Availability statement to reflect the information you provide*

Although we stated we would make the Data fully available without restriction, they were gathered from a national database, from the Portuguese Society of Cardiology, and we are not authorized to provide them for a repository. We were not aware of this at the moment of the submission, however, we believe in open science and therefore we would like to change our Data Availability statement to the option that allows sharing the data sets available only upon request and we will make efforts to assure access.

*4. Please review your reference list to ensure that it is complete and correct. If you have cited papers that have been retracted, please include the rationale for doing so in the manuscript text, or remove these references and replace them with relevant current references. Any changes to the reference list should be mentioned in the rebuttal letter that accompanies your revised manuscript. If you need to cite a retracted article, indicate the article’s retracted status in the References list and also include a citation and full reference for the retraction notice.*

Reference list was reviewed and no changes were made.

Response to Reviewers

We thank the kind comments from the reviewers, which made us proud of our work.

Reviewer 1 made an important comment in order to improve the manuscript, suggesting “including data on hospital mortality as well, in case the authors have collected data on that.” We totally agree with this comment, but as matter of fact these data were not collected. Nevertheless we included this suggestion in a new section named Limitations and Future directions.

Reviewer 2 made also an important comment in order to be included in future papers suggesting “performing analysis of the different clinical presentations of coronary syndromes in addition to those with ST elevation”. This suggestion was also included in the new section named Limitations and Future directions.
